# What MRI-based tumor size measurement is best for predicting long-term survival in uterine cervical cancer?

**DOI:** 10.1186/s13244-022-01239-y

**Published:** 2022-06-17

**Authors:** Njål Lura, Kari S. Wagner-Larsen, David Forsse, Jone Trovik, Mari K. Halle, Bjørn I. Bertelsen, Øyvind Salvesen, Kathrine Woie, Camilla Krakstad, Ingfrid S. Haldorsen

**Affiliations:** 1 Department of Radiology, Mohn Medical Imaging and Visualization Centre, Haukeland University Hospital, Jonas Lies vei 65, 5021 Bergen, Norway; 2 Section for Radiology, Department of Clinical Medicine, University of Bergen, Bergen, Norway; 3 Department of Obstetrics and Gynecology, Haukeland University Hospital, Bergen, Norway; 4 Department of Clinical Science, Centre for Cancer Biomarkers, University of Bergen, Bergen, Norway; 5 Department of Pathology, Haukeland University Hospital, Bergen, Norway; 6 Clinical Research Unit, Department of Clinical and Molecular Medicine, Norwegian University of Science and Technology, Trondheim, Norway

**Keywords:** MRI, Cervical cancer, Tumor size, Disease-specific survival

## Abstract

**Background:**

Tumor size assessment by MRI is central for staging uterine cervical cancer. However, the optimal role of MRI-derived tumor measurements for prognostication is still unclear.

**Material and methods:**

This retrospective cohort study included 416 women (median age: 43 years) diagnosed with cervical cancer during 2002–2017 who underwent pretreatment pelvic MRI. The MRIs were independently read by three radiologists, measuring maximum tumor diameters in three orthogonal planes and maximum diameter irrespective of plane (MAX_imaging_). Inter-reader agreement for tumor size measurements was assessed by intraclass correlation coefficients (ICCs). Size was analyzed in relation to age, International Federation of Gynecology and Obstetrics (FIGO) (2018) stage, histopathological markers, and disease-specific survival using Kaplan–Meier-, Cox regression-, and time-dependent receiver operating characteristics (tdROC) analyses.

**Results:**

All MRI tumor size variables (cm) yielded high areas under the tdROC curves (AUCs) for predicting survival (AUC 0.81–0.84) at 5 years after diagnosis and predicted outcome (hazard ratios [HRs] of 1.42–1.76, *p* < 0.001 for all). Only MAX_imaging_ independently predicted survival (HR = 1.51, *p* = 0.03) in the model including all size variables. The optimal cutoff for maximum tumor diameter (≥ 4.0 cm) yielded sensitivity (specificity) of 83% (73%) for predicting disease-specific death after 5 years. Inter-reader agreement for MRI-based primary tumor size measurements was excellent, with ICCs of 0.83–0.85.

**Conclusion:**

Among all MRI-derived tumor size measurements, MAX_imaging_ was the only independent predictor of survival. MAX_imaging_ ≥ 4.0 cm represents the optimal cutoff for predicting long-term disease-specific survival in cervical cancer. Inter-reader agreement for MRI-based tumor size measurements was excellent.

**Supplementary Information:**

The online version contains supplementary material available at 10.1186/s13244-022-01239-y.

## Key points

- Maximum tumor size is the only size measurement independently predicting disease-specific survival.
- Maximum tumor size ≥ 4.0 cm represents an optimal cutoff for predicting reduced survival.
- Inter-reader reproducibility for MRI-based tumor size measurements is excellent in cervical cancer.

## Introduction

Uterine cervical cancer is the fourth most common cancer in women worldwide and one of the leading causes of cancer-related deaths, especially among women living in low-income countries [1]. Primary maximum tumor size is incorporated in the recently updated 2018 International Federation of Gynecology and Obstetrics (FIGO) staging system and defines stages IB1 (≤ 2 cm), IB2 (> 2 cm and ≤ 4 cm), and IB3 (> 4 cm) in tumors confined to the cervix and stages IIA1 (≤ 4 cm) and IIA2 (> 4 cm) in tumors involving the upper two-thirds of the vagina [2]. The 2018 FIGO guidelines recommend dual pretreatment staging by TNM and include imaging findings in the FIGO staging system [3].

Studies assessing the prognostic impact of primary tumor size based on clinical pelvic examination [4–6] or tumor measurements in surgical specimens [7–10] uniformly report large tumor size to predict poor survival in cervical cancer. Histopathologic tumor size reportedly also predicts prognosis across FIGO stages I–IV [9]. However, histopathologic tumor size assessments require access to surgical specimen, which is first available after surgical resection and only in the subgroup of patients receiving primary surgical treatment.

Pelvic magnetic resonance imaging (MRI) is often employed at primary diagnostic work-up for local staging in cervical cancer and guides the choice of primary treatment, primary surgery being standard for small lesions confined to the cervix and chemoradiotherapy/brachytherapy in locally advanced disease. Although MRI-assessed large tumor size is reportedly associated with microscopic lymphovascular space invasion, parametrial invasion, metastatic pelvic lymph nodes [11–13], and poor outcome [14–16], the optimal role of different MRI-derived tumor size measurements for pretreatment prognostication in cervical cancer is not defined. Furthermore, only a few studies have reported inter-reader reproducibility metrics for tumor size measurements by MRI in cervical cancer [17, 18].

The aim of this study was to explore the value of different MRI-based tumor size measurements for the prediction of long-term disease-specific survival in cervical cancer and to assess the inter-reader reproducibility for these tumor size measurements in a large cervical cancer patient cohort.

## Material and methods

### Patient overview

**Fig. 1:**
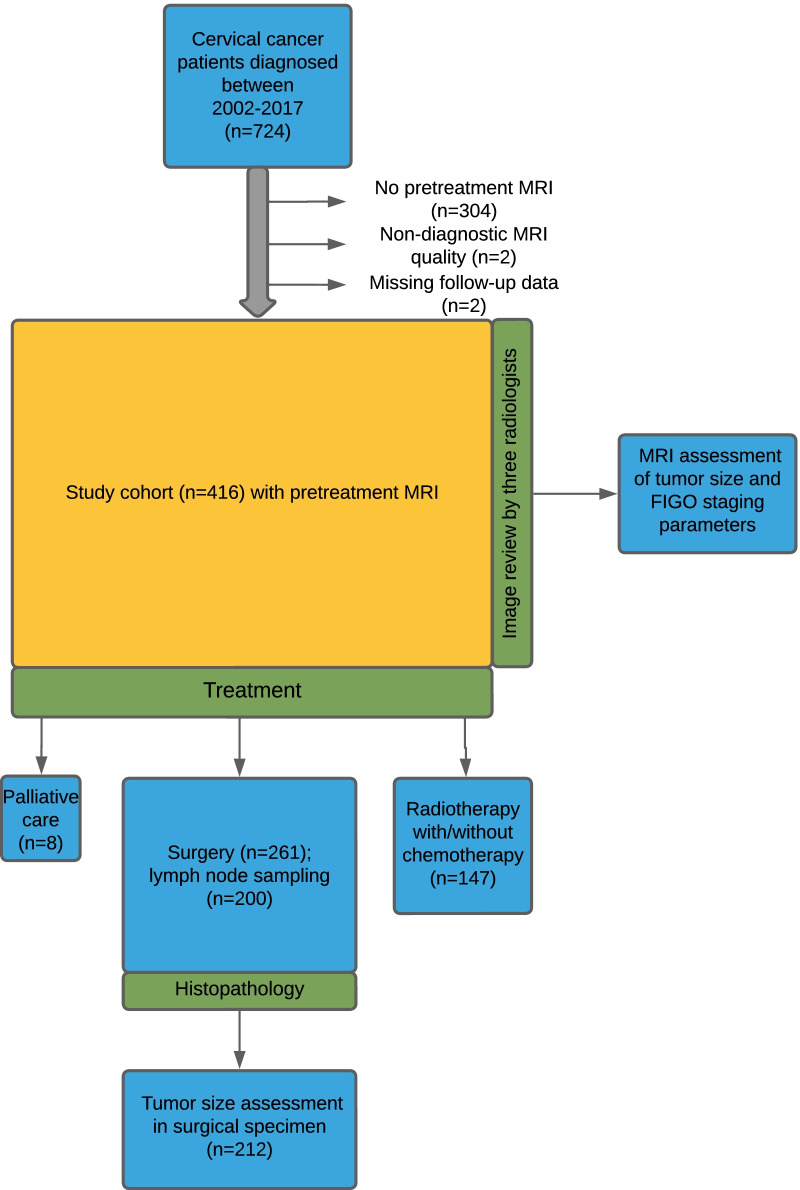
Flowchart illustrating patient inclusion and study setup, including MRI review with tumor size measurements and histological assessment of tumor size in a subgroup of surgically treated patients

This study was conducted under institutional review board-approved protocols with informed, written consent from all patients. Out of 724 histologically confirmed cervical cancer patients treated at the same university hospital (serving a population of ~ 1 million inhabitants) between May 2002 and December 2017, a total of 420 patients underwent pretreatment pelvic MRI. After excluding patients with incomplete MRI (*n* = 2) or missing follow-up data (*n* = 2), the study cohort comprised 416 cervical cancer patients (Fig. 1). The MRI study cohort and the entire patient cohort had similar clinical and histological characteristics (Additional file 2: Table S1).

Clinical patient data (age, clinical tumor size) were recorded. All patients were classified according to FIGO (2009) staging system and reclassified according to FIGO (2018) [2]. The histological cervical cancer diagnosis was established based on formalin-fixed, paraffin-embedded tissue samples from cervical biopsies. Information about histological type and grade was retrospectively collected based on routine histopathology evaluation at primary diagnostic work-up and from expert pathologist review [10]. Primary treatment- and follow-up data regarding disease-specific survival were collected from patient records (last accessed September 2021) and from correspondence with the responsible gynecologist.

Histopathological maximum tumor size (MAX_histology_) was recorded when reported in the pathology report (*n* = 212); both macroscopic (macro-MAX_histology_) and microscopic (micro-MAX_histology_) tumor size was reported in 18/212 (8%), only micro-MAX_histology_ in 140/212 (66%) and only macro-MAX_histology_ in 54/212 (25%). The variable MAX_histology_ used microscopic tumor size when this was reported and macroscopic tumor size in cases without microscopic measurement. In patients treated with primary hysterectomy (*n* = 224), the median time from MRI examination to surgery was 14 days (IQR 7–25 days). The median (interquartile range [IQR]) follow-up time for all patients was 82 (55–115) months and 91 (65–127) months for survivors.

### MRI protocol

The MRI examinations were performed at different hospitals with scanners from Siemens Healthineers/ GE Healthcare)/Philips Healthcare in 250/13/153 patients and on 1.5 T/3.0 T systems in 329/87 patients. The imaging protocols included pelvic sagittal and axial oblique (perpendicular to the long axis of the uterine cervix) T2-weighted images in all and axial T1-weighted gradient-echo images in 95% (397/416) of the examinations, among which 10% (40/397) included series with intravenous contrast. Pelvic diffusion-weighted imaging was included in the protocol in 66% (273/416).

### Data analysis

**Fig. 2:**
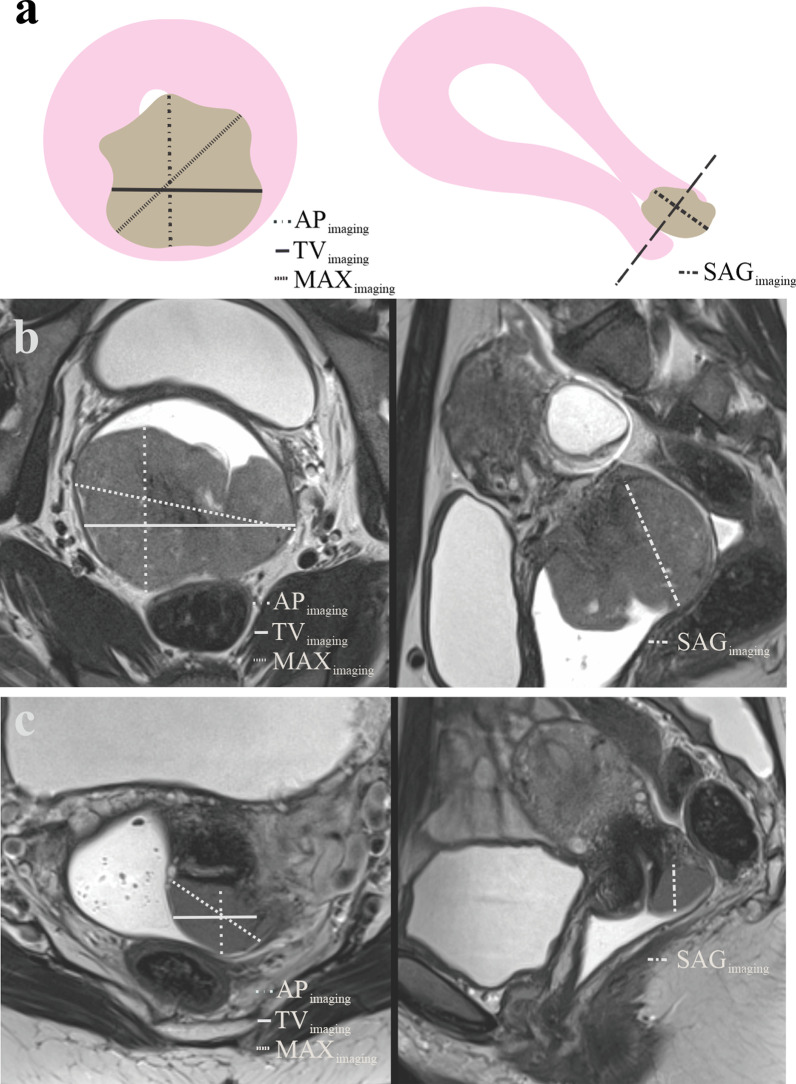
Graphical illustration of the uterus (**a**) in the axial oblique (perpendicular to the long axis of the cervix; left) and sagittal plane (right) with a tumor (brown) invading the cervical stroma but confined to the uterine cervix. T2-weighted MRI in the same planes in a 37-year-old patient (FIGO 2018) stage IB2 squamous cell carcinoma (**b**) and a 23-year-old patient (FIGO 2018) stage IIB squamous cell carcinoma (**c**) depicts hyperintense cervical tumors. Tumor size was measured as the largest anteroposterior (AP_imaging_) and transverse (TV_imaging_) diameters in the axial oblique plane, largest diameter parallel to the long axis of the cervical lumen in the sagittal plane (SAG_imaging_) and maximum tumor diameter (MAX_imaging_) irrespective of the plane

All images were deidentified and read independently by three radiologists (reader 1 = N.L., reader 2 = K.W.L., and reader 3 = I.J.M.) having 5, 10, and 20 years of experience, respectively, with reading pelvic MRI. The readers were blinded for clinical data, histological diagnosis, and patient outcome. The readers reviewed the images independently and measured the largest anteroposterior (AP_imaging_)- and transverse (TV_imaging_)-tumor diameters in the axial oblique plane and the largest diameter parallel to the long axis of the cervical lumen in the sagittal plane (SAG_imaging_) and maximum diameter irrespective of the plane (MAX_imaging_) on T2-weighted series (Fig. 2). When available, the diffusion-weighted imaging sequences were used to support the placement of tumor measurements on T2 images. In patients with no visible tumor on MRI (in 35% [146/416]), tumor size was recorded with the numerical value “0.” All readers also recorded whether MRI indicated tumor growth into the vagina, parametrium, or rectum/bladder or presence of enlarged (short-axis diameter > 10 mm) pelvic lymph nodes.

To establish the overall imaging findings based on the recordings by all three readers, we computed a new data set named “consensus variables” in which the value given by the majority of readers was recorded for categorical variables, and the median value was recorded for continuous variables (e.g., tumor size measurements). Tumor volumes (TVOL_imaging_) were calculated from consensus variables using the following equation: ${\mathrm{TVOL}}_{\mathrm{imaging}}=4/3\pi ({\mathrm{AP}}_{\mathrm{imaging}}/2\times {\mathrm{TV}}_{\mathrm{imaging}}/2\times {\mathrm{SAG}}_{\mathrm{imaging}}/2$).

### Statistical analysis

All tumor size variables had non-normal distributions (Shapiro–Wilk test, *p* < 0.001 for all). Tumor size was analyzed in relation to clinical and histopathologic characteristics and other imaging findings (i.e., vaginal tumor extension, parametrial infiltration, enlarged lymph nodes or bladder or rectal invasion) using Mann–Whitney U test for two categories, Kruskal–Wallis H test or Jonckheere–Terpstra trend test for multiple categories and linear regression for continuous variables.

Correlation between tumor size variables was analyzed using Spearman's rank correlation test. Disease-specific survival was analyzed in relation to tumor size variables and clinicopathological variables using Cox regression analysis and Kaplan–Meier with the log-rank test. All variables in the Cox regression analyses satisfied the assumption of proportional hazard (the Schoenfeld test of residuals, *p* ≥ 0.35). Multiple imputations were performed using the “mice”-algorithm [19] for missing values in Cox regression analysis. The “fastbw”-function in the “rms” R-package [20] was used for variable selection in the multivariable Cox regression analysis.

Time-dependent receiver operating characteristic (tdROC) [21, 22] analyses were used to evaluate and compare the diagnostic performance of tumor measurements for predicting disease-specific survival. The “timeROC” R-package [23] was used for calculating AUC at specific time points. Optimal cutoffs with corresponding time-dependent sensitivity and specificity for tumor size for predicting disease-specific survival were determined by tdROC curve at 5 years after diagnosis selecting the highest Youden index [24, 25]. The integral of AUC (iAUC) from time points with events for 10 years after primary diagnosis was calculated using the “risksetROC” R-package [26].

Inter-reader agreement for tumor size measurements was assessed by intraclass correlation coefficient ICC analysis and classified as poor (ICC = 0–0.39), fair (0.40–0.59), good (0.60–0.74), or excellent (0.75–1.00) [27]. A comparison between MAX_imaging_ and MAX_histology_ was performed using Bland–Altman plots. All comparisons of tdROC analyses were performed using bootstrapping with resampling 10,000 times. *p* values involving either multiple comparisons of variable means, ROC analysis, Schoenfeld test, and Cox regression were adjusted using Holm–Bonferroni corrections separately [28, 29]. The data were analyzed using R software (version 4.0.3, R Foundation for Statistical Computing, Vienna, Austria) [30]. All reported *p* values were two-sided and considered significant when below 0.05.

## Results

### Patients and treatment

Median (IQR) patient age at primary diagnosis in the study cohort (*n* = 416) was 43 (36–55) years. In total, 63% (260/416) of the patients were diagnosed with FIGO (2018) stage I, 12% (50/416) with stage II, 19% (81/416) with stage III, and 6% (25/416) with stage IV. Primary treatment consisted of only surgery in 51% (210/416), primary surgery followed by adjuvant therapy in 12% (51/416), primary radiotherapy with/without chemotherapy in 35% (147/416), and palliative chemotherapy/supportive care in 2% (8/416). Primary surgical treatment consisted of radical hysterectomy (*n* = 199), simple hysterectomy (*n* = 25), cervical amputation (*n* = 1) (surgical procedure in these 225 patients: laparotomy (*n* = 191), robot-assisted laparoscopy (*n* = 25), or conventional laparoscopy (*n* = 9)), conization (*n* = 31), or fertility-sparing surgery (*n* = 5) (Table 1).

**Table 1:** MRI-assessed maximum tumor diameter (MAX_imaging_) and tumor volume (TVOL_imaging_) in relation to clinical and histological characteristics in 416 patients with cervical cancer

Variable	*N*	MAX_imaging_, cm median (IQR)	*P*	TVOL_imaging_, cl median (IQR)	*p*
FIGO (2018) stage (*n* = 416)			< 0.001***		< 0.001***
I	260	0 (0–3.0)		0 (0–0.5)	
II	50	4.7 (3.8–6.0)		2.4 (1.2–5.0)	
III	81	5.4 (4.6–6.5)		4.0 (1.9–6.5)	
IV	25	6.2 (4.6–7.6)		6.0 (2.4–9.6)	
Clinical tumor size (cm) (*n* = 230)			< 0.001***		< 0.001***
< 2	46	1.4 (0–3.2)		0.1 (0–0.7)	
2–4	109	3.8 (2.9–4.6)		1.0 (0.4–2.6)	
> 4	75	5.6 (4.7–6.7)		4.4 (2.3–7.0)	
Primary treatment (*n* = 416)			< 0.001**		< 0.001**
Surgery only^a^	210	0 (0–2.1)		0 (0–0.2)	
Surgery^a^ and adjuvant treatment	51	3.3 (1.5–4.5)		1.0 (0.1–2.2)	
Primary radiotherapy with or without chemotherapy	147	5.1 (4.2–6.3)		3.3 (1.4–6.1)	
Other^b^	8	7.2 (6.0–8.6)		6.4 (5.1–15.9)	
Histologic subtype (*n* = 416)			0.03**		0.03**
Adenocarcinoma	92	2.1 (0–4.0)		0.2 (0–1.2)	
Squamous cell carcinoma	292	3.2 (0–4.9)		0.6 (0–3.1)	
Other^c^	32	3.8 (0–5.4)		1.1 (0–4.7)	
Histologic grade (*n* = 343)			< 0.001*		< 0.001*
Low/medium	253	3.0 (0–4.7)		0.4 (0–2.5)	
High	90	4.3 (1.8–5.6)		1.6 (0.1–4.5)	
Parametrial infiltration (*n* = 205)			0.006*		0.006*
No	200	0 (0–2.7)		0 (0–0.4)	
Yes	5	4.8 (3.0–6.9)		3.0 (0.5–6.4)	
Lymph node metastasis (*n* = 200)			0.02*		0.02*
No	177	1.1 (0–3.0)		0.03 (0–0.5)	
Yes	23	3.0 (0–4.6)		0.5 (0–2.1)	

	MAX_imaging_			TVOL_imaging_^d^		
Dependent variables	*R*^2^	*β*	*P*	*R*^2^	*Β*	*p*
Linear regression for continuous variables^e^						
Age (decade) (*n* = 416)	0.10	0.62	** < 0.001**	0.11	0.17	** < 0.001**
Histopathological MAX_histology_(cm) (*n* = 212)	0.73	0.82	**< 0.001**	0.69	0.20	** < 0.001**
Microscopic depth of invasion (cm) (*n* = 181)	0.41	2.75	** < 0.001**	0.43	0.79	** < 0.001**

FIGO, International Federation of Gynecology and Obstetrics; IQR, interquartile range; MAX_histology_, maximum histological tumor diameter

*P* values corrected for multiple testing of each size variable by Holm–Bonferroni method. Significant *p* values are given in boldface

^*^Mann–Whitney U test

^**^Kruskal–Wallis H test

^***^Jonckheere–Terpstra trend test

^a^Primary surgical treatment in 225 patients consisted of radical hysterectomy (*n* = 199), simple hysterectomy (*n* = 25), and cervical amputation (*n* = 1) (surgical procedure: laparotomy (*n* = 191), robot-assisted laparoscopy (*n* = 25), or conventional laparoscopy (*n* = 9)), whereas 36 patients were surgically treated with conization (*n* = 31) or fertility-sparing surgery (*n* = 5)

^b^Palliation (*n* = 1) or only chemotherapy (*n* = 7)

^c^Adenosquamous (*n* = 14), neuroendocrine (*n* = 9), or undifferentiated carcinoma (*n* = 9)

^d^The third root of the tumor volume

^e^Linear models with MAX_imaging_ or TVOL_imaging_ as dependent variables

### Large tumor size is associated with aggressive clinicopathological characteristics

In 35% (146/416) of the patients (among whom 73% (107/146) had undergone conization prior to MRI), no measurable tumor (recorded as 0 cm) was visible. In the total study cohort, the different MRI tumor size measurements had a median (IQR) of 2.0 (0–3.7) cm for AP_imaging_, 2.4 (0–3.9) cm for TV_imaging_, 1.7 (0–3.7) cm for SAG_imaging_, 2.8 (0–4.8) cm for MAX_imaging_, and 0.4 (0–2.6) cl for TVOL_imaging_ (Table 2). All tumor size measurements were strongly positively correlated (*r* = 0.95–0.99; *p* < 0.001 for all) (Table 2).

**Table 2:** Median (IQR) values for MRI-assessed tumor size variables and their correlations in 416 patients with cervical cancer

	AP_imaging_	TV_imaging_	SAG_imaging_	MAX_imaging_	TVOL_imaging_
Median (IQR)	2.0 (0–3.7) cm	2.4 (0–3.9) cm	1.7 (0–3.7) cm	2.8 (0–4.8) cm	0.4 (0–2.6) cl
	*r*	*r*	*r*	*r*	*r*
AP_imaging_	1	0.97*	0.95*	0.98*	0.98*
TV_imaging_		1	0.95*	0.98*	0.98*
SAG_imaging_			1	0.97*	0.98*
MAX_imaging_				1	0.99*
TVOL_imaging_					1

AP, Anteroposterior diameter; IQR, interquartile range; MAX_imaging_, maximum diameter (irrespective of plane); SAG_imaging_, sagittal diameter; TV_imaging_, transverse diameter; and TVOL_imaging_, tumor volume

*r* = Spearman's rank correlation coefficient (rho)

^*^Correlation is significant, *p* < 0.001 (2-tailed)

Large AP_imaging_, TV_imaging_, SAG_imaging_, MAX_imaging,_ and TVOL_imaging_ were all associated with higher age, higher FIGO (2018) stage, squamous cell carcinoma subtype, high-grade histology, histopathologically verified parametrial infiltration, lymph node metastasis, and microscopic depth of invasion (*p* ≤ 0.03 for all; figures for MAX_imaging_ and TVOL_imaging_ presented in Table 1). Larger MAX_imaging_ and TVOL_imaging_ were also associated with positive MRI findings for vaginal tumor extension (in 42% [173/416]), parametrial infiltration (in 43% [180/416]), enlarged lymph nodes (in 14% [59/416]), or tumor invasion into the bladder/rectum (9% [36/416]) (*p* < 0.001 for all; Table 3).

**Table 3:** MRI-assessed maximum tumor diameter (MAX_imaging_) and tumor volume (TVOL_imaging_) in relation to other imaging findings in 416 patients with cervical cancer

Imaging findings	*n*	MAX_imaging_, cm median (IQR)	p*	TVOL_imaging_, cl median (IQR)	p*
Vaginal tumor extension			** < 0.001**		** < 0.001**
No	243	0 (0–2.2)		0 (0–0.2)	
Yes	173	4.9 (4.0–6.2)		3.0 (1.2–6.0)	
Parametrial infiltration			** < 0.001**		** < 0.001**
No	236	0 (0–1.9)		0 (0–0.1)	
Yes	180	4.9 (4.1–6.1)		3.0 (1.4–6.0)	
Enlarged (> 1 cm) pelvic lymph nodes			** < 0.001**		** < 0.001**
No	357	2.0 (0–4.2)		0.2 (0–1.6)	
Yes	59	5.6 (4.9–6.6)		4.9 (2.5–6.8)	
Tumor invasion into the bladder or rectum			** < 0.001**		** < 0.001**
No	380	2.4 (0–4.5)		0.24 (0–2.0)	
Yes	36	6.6 (5.1–8.0)		6.2 (4.0–9.8)	

IQR, Interquartile range

*P* values corrected for multiple testing of each size variable by Holm–Bonferroni method. Significant *p* values are given in boldface

*Mann–Whitney U test for two categories

### Large tumor size predicts poor survival in cervical cancer

All tumor size measurements (AP_imaging_, TV_imaging_, SAG_imaging_, MAX_imaging_ (cm)) predicted disease-specific survival with hazard ratios (HRs) ranging from 1.42 to 1.76 (*p* < 0.001 for all; Table 4). In a multivariable model including all tumor size measurements, only MAX_imaging_ independently predicted disease-specific survival with HR (95% CI) of 1.51 (1.11–2.04; *p* = 0.03) (Table 4). In a multivariable model including MAX_imaging_ (cm), age (decade), and FIGO (2018) stage (III/IV vs. I/II), all variables independently predicted disease-specific survival, yielding HRs (95% CI) of 1.27 (1.18–1.39; *p* < 0.001), 1.57 (1.34–1.85; *p* < 0.001), and 3.24 (1.88–5.59; *p* < 0.001), respectively (Table 4). Subgroup analyses for different FIGO (2018) stages found that MAX_imaging_ (cm) significantly predicted disease-specific survival for FIGO stage I (HR = 1.59, 95% CI 1.15–2.20, *p* = 0.01) and stage III (HR = 1.32, 95% CI 1.16–1.51, *p* < 0.001), whereas not for stage II (HR = 1.15, 95% CI 0.83–1.61, *p* = 0.40) and stage IV (HR = 1.11, 95% CI 0.97–1.28, *p* = 0.28) (Table 4). Similar results were observed for predicting recurrence- or progression-free survival (Additional file 2: Table S3).

**Table 4:** Uni- and multivariable hazard ratios for MRI-derived tumor size variables for predicting disease-specific survival in 416 patients with uterine cervical cancer (78 patients died from disease)

Variables	Univariable HR (95% CI)	p*	Multivariable HR (95% CI)^a^	p*
AP_imaging_ (cm)	1.76 (1.58–1.97)	** < 0.001**	1.11 (0.78–1.58)	0.77
TV_imaging_ (cm)	1.69 (1.53–1.87)	** < 0.001**	1.14 (0.85–1.54)	0.77
SAG_imaging_ (cm)	1.42 (1.33–1.51)	** < 0.001**	0.82 (0.65–1.04)	0.28
MAX_imaging_ (cm)	1.44 (1.35–1.53)	** < 0.001**	1.51 (1.11–2.04)	**0.03**

Variables	Univariable HR (95% CI)	p*	Multivariable HR (95% CI)^a^	p*
MAX_imaging_ (cm)	1.44 (1.35–1.53)	** < 0.001**	1.27 (1.18–1.39)	** < 0.001**
Age (decade)	1.69 (1.48–1.93)	** < 0.001**	1.57 (1.34–1.85)	** < 0.001**
FIGO (2018) stage (III/IV vs. I/II)	8.64 (5.36–13.93)	** < 0.001**	3.24 (1.88–5.59)	** < 0.001**

MAX_imaging_ (cm) in FIGO (2018) subgroups	Univariable HR (95% CI)	p*	–	–
FIGO I (*n* = 260)	1.59 (1.15–2.20)	**0.01**	-	-
FIGO II (*n* = 50)	1.15 (0.83–1.61)	0.40	–	–
FIGO III (*n* = 81)	1.32 (1.16–1.51)	** < 0.001**	–	–
FIGO IV (*n* = 25)	1.11 (0.97–1.28)	0.28	–	–
Subgroup analysis of surgically treated patients who had histopathological assessments of primary tumor (*n* = 261; 20 patients died from disease) with pelvic lymph node sampling (*n* = 200 patients; 17 patients died from disease)^b^				

	Univariable HR (95% CI)	p*	Multivariable HR (95% CI)^c^	p*
MAX_imaging_ (cm)	1.77 (1.39–2.26)	** < 0.001**	1.61 (1.25–2.08)	** < 0.001**
Age (decade)	1.65 (1.21–2.24)	**0.002**	–	–
MAX_histology_ (cm)	1.49 (1.25–1.78)	** < 0.001**	–	–
Inflammatory reaction (yes vs. no)	0.68 (0.23–2.04)	0.50	–	–
Microscopic depth of invasion (cm)	3.30 (1.40–7.79)	**0.006**	–	–
Vascular space invasion (yes vs. no)	5.73 (2.33–14.05)	** < 0.001**	–	–
Histologic grade (high vs. low/medium)	5.67 (2.31–13.86)	** < 0.001**	–	–
Lymph node metastasis (yes vs. no)	13.79 (5.70–33.41)	** < 0.001**	4.54 (1.67–12.32)	** < 0.001**

AP_imaging_, Anteroposterior tumor diameter at MRI; CI, confidence interval; FIGO, International Federation of Gynecology and Obstetrics; HR, hazard ratio; MAX_imaging_, maximum tumor diameter at MRI; MAX_histology,_ maximum tumor diameter in histological samples; MRI, magnetic resonance imaging; SAG_imaging_, sagittal tumor diameter at MRI; and TV_imaging_, transverse tumor diameter at MRI

*Cox proportional hazards model; all *p* values corrected for multiple testing with Holm–Bonferroni method. Significant *p* values are given in boldface

^a^Includes all variables listed

^b^Missing data were handled by multiple imputation in order to perform multivariable analysis on all patients treated with surgery

^c^Variables in model were selected by using the “fastbw”-function in the “rms” r-package (1)

Among the surgically treated patients (*n* = 261) with detailed histopathological assessments, MAX_imaging_, patient age, MAX_histology_, microscopic depth of invasion, vascular space invasion, histological grade, and lymph node metastasis (histologically verified), all predicted disease-specific survival in univariable analyses. However, only MAX_imaging_ and lymph node metastasis independently predicted survival in the multivariable model (Table 4). The same findings were observed for predicting recurrence- or progression-free survival (Additional file 2: Table S3).

**Fig. 3:**
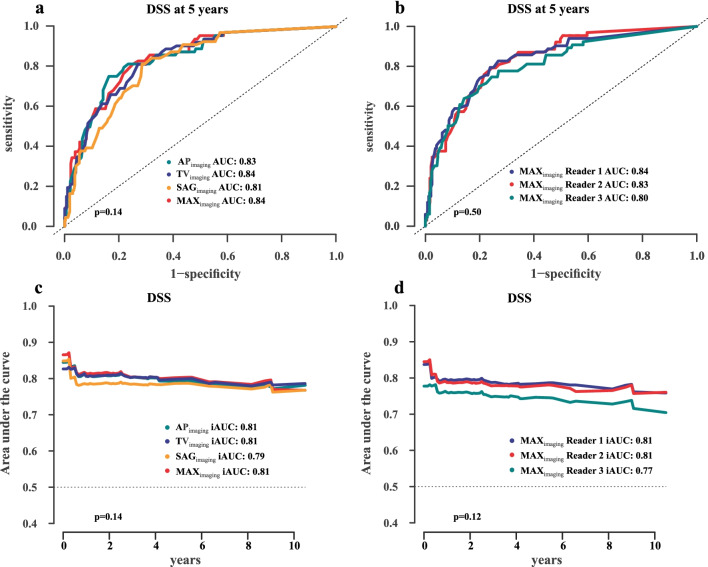
Time-dependent receiver operating characteristic curves (tdROC) at 5 years after diagnosis (**a**, **b**) and integral of area under the time-dependent ROC curve (iAUC) for 10 years after diagnosis (**c**, **d**) for predicting disease-specific survival (DSS) based on the MRI-derived tumor size measurements (**a**, **c**) and for MAX_imaging_ for the three readers (**b**, **d**). All tumor measurements yielded high and similar areas under the tdROC curves (AUC = 0.81–0.84; *p* = 0.14) (**a**) and iAUC (iAUC = 0.79–0.81; *p* = 0.14) (**c**) for predicting DSS. MAX_imaging_ for the three readers yielded similarly high areas under the tdROC curves (AUC = 0.80–0.84; *p* = 0.50) (**b**) and iAUC (iAUC = 0.77–0.81; *p* = 0.12) (**d**) for predicting DSS

**Fig. 4:**
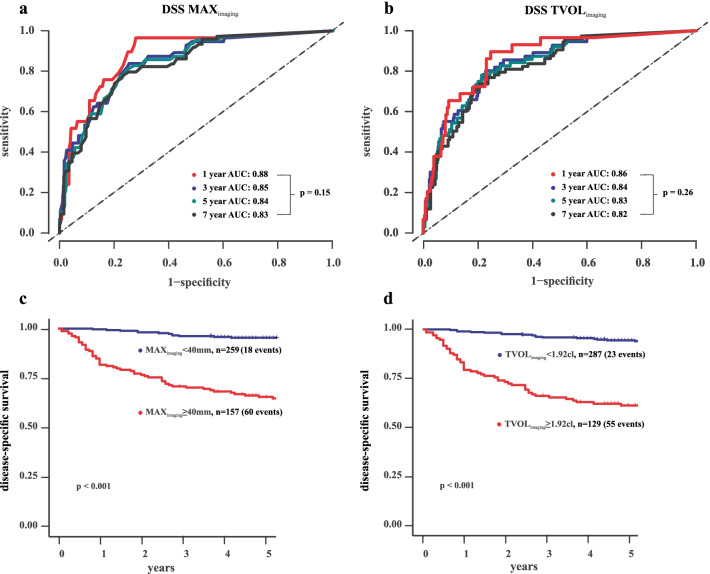
Time-dependent receiver operating characteristics curves (tdROC) at 1, 3, 5, and 7 years after primary diagnosis for predicting disease-specific survival (DSS) based on MRI-measured maximum tumor diameter (MAX_imaging_) (**a**) and tumor volume (TVOL_imaging_) (**b**); both showed non-significant reduction in AUC over time. Kaplan–Meier curves depict significantly reduced disease-specific survival in patients with MAX_imaging_ ≥ 4.0 cm (*p* < 0.001) (**c**) and TVOL ≥ 1.92 cl (*p* < 0.001) (**d**). For each category: number of patients/number of patients dying from the disease

All tumor size variables yielded a similarly high area under the tdROC curve for predicting disease-specific survival at 5 years (AUCs of 0.81–0.84; *p* = 0.14) (Fig. 3a) and 10 years (iAUC in the range of 0.79–0.81; *p* = 0.14) (Fig. 3c) after primary diagnosis. Areas under the tdROC curve for predicting disease-specific survival were stable over time with no significant difference at year 1 and year 7 for MAX_imaging_ (AUC = 0.88 and 0.83, respectively; *p* = 0.15) (Fig. 4a) and TVOL_imaging_ (AUC = 0.86 and 0.82, respectively; *p* = 0.26) (Fig. 4b).

Optimal cutoffs (with corresponding time-dependent sensitivity [specificity]) for the tumor size measurements for predicting disease-specific death at 5 years were: AP_imaging_ ≥ 3.0 cm (80% [73%]), TV_imaging_ ≥ 3.8 cm (74% [83%]), SAG_imaging_ ≥ 2.3 cm (85% [69%]), MAX_imaging_ ≥ 4.0 cm (82% [73%]) , TVOL_imaging_ ≥ 1.92 cl (76% [78%]). Overall disease-specific survival at 5 years for patients with MAX_imaging_ ≥ 4.0/ < 4 cm and with TVOL_imaging_ ≥ 1.92/ < 1.92 cl was 65%/95% and 60%/94%, respectively (Fig. 4c, d).

### Comparison between histological tumor size and MRI-measured tumor size

**Fig. 5:**
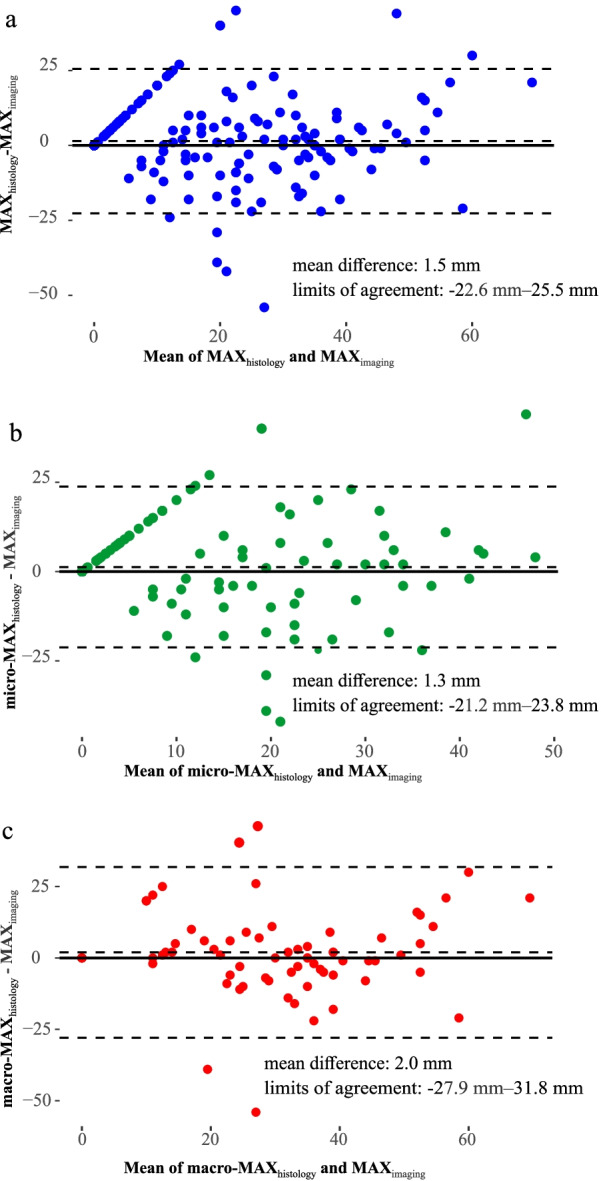
Bland–Altman plots depicting the difference in histological tumor size and MRI-assessed tumor size: for MAX_histology_ and MAX_imaging_ (*n* = 212) (**a**), tumor size based on microscopy (micro-MAX_histology_) and MAX_imaging_ (*n* = 158) (**b**) and tumor size based on macroscopic assessment (macro-MAX_histology_) and MAX_imaging_ (*n* = 72) (**c**). Mean MAX_histology_ was 1.5 mm larger than MAX_imaging_ (**a**), mean micro-MAX_histology_ was 1.3 mm larger than MAX_imaging_ (**b**), and mean macro-MAX_histology_ was 2.0 mm larger than MAX_imaging_ (**c**)

In the subgroup of patients having histological tumor size assessment (*n* = 212), mean MAX_histology_ (mean = 17.4 mm) was 1.5 mm higher than MAX_imaging_ (mean = 15.9 mm) (Fig. 5a), yielding an ICC of 0.77 (95%CI 0.67–0.85) for the two tumor size measurements. Mean micro-MAX_histology_ was 1.3 mm larger than MAX_imaging_ (Fig. 5b), whereas macro-MAX_histology_ was 2.0 mm larger than MAX_imaging_ (Fig. 5c). MAX_histology_ and MAX_imaging_ yielded similar AUCs of 0.76 and 0.83, respectively, for predicting DSS at 5 years (*p* = 0.38) (Additional file 1: Figure S1).

### Excellent and good inter-reader reproducibility of tumor size measurements

The three readers' inter-reader reproducibility for tumor size measurements was excellent, with ICCs of 0.83–0.85 when including all patients in the analysis, and good with ICCs of 0.69–0.73 when analyzing only patients with visible tumor (Additional file 2: Table S2). The tdROC analyses for the three readers for prediction of disease-specific survival at 5 years by MAX_imaging_ yielded a similarly high area under the ROC curves (AUC = 0.80–0.84; *p* = 0.50) (Fig. 3b). Similarly, the iAUC was high (0.77–0.81) with no significant difference across readers (*p* = 0.12) (Fig. 3d).

## Discussion

The purpose of this study was to explore the value of MRI-derived tumor size markers for predicting long-term disease-specific survival and assess the inter-reader reproducibility for tumor size measurements at MRI in cervical cancer patients. We show that MRI-measured maximum diameter (MAX_imaging_) was the only size measurement independently predicting disease-specific survival in a multivariable model including all size variables, and that maximum diameter yielded high diagnostic performance (AUC of 0.84) for predicting disease-specific survival after 5 years. Furthermore, we demonstrate excellent inter-reader reproducibility for maximum diameter measurements (ICC = 0.85) based on MRI.

This study found that the tumor size measurements were all highly correlated (Spearman's rank correlation coefficient: 0.95–0.99) and that all had a significant impact on disease-specific survival. However, in the multivariable analyses, MAX_imaging_ was the only size variable independently predicting disease-specific survival. The superiority of MAX_imaging_ may be related to it being measured irrespective of planes and thus not limited to specific directions, which is the case for the other size measurements in the standardized planes (anteroposterior, transverse, and sagittal). Interestingly, the finding that MAX_imaging_ yields the highest prognostic power also supports current 2018 FIGO guidelines, where the maximum diameter is the only tumor size measurement included in the staging system. Furthermore, the optimal cutoff for MAX_imaging_ identified in the present study (≥ 4.0 cm) based on its prognostic power at 5 years after primary cervical cancer diagnosis is almost identical to the maximum diameter cutoff (> 4.0 cm) used in 2018 FIGO for discriminating IB3 from IB2 and IIA2 from IIA1 [2].

In the present study, large tumor size was associated with histopathologically verified parametrial infiltration and lymph node metastasis in surgically treated patients, as well as with positive corresponding MRI findings guiding stage assignment in 2018 FIGO staging system [2]. This is in line with prior studies reporting associations between large tumor size (both histopathological and MRI-measured) and pathologically enlarged lymph nodes on MRI [31], as well as with histologically verified lymph node metastases or parametrial infiltration [11–13].

In the subgroup analyses of FIGO (2018) stages, MAX_imaging_ significantly predicted disease-specific survival for FIGO stages I and III (*p* ≤ 0.01 for both) but not for FIGO stages II and IV. Similar to this, a large cervical cancer patient (*n* = 18,649) study has reported that tumor size assessed in hysterectomy specimens predicted disease-specific survival within FIGO (1988/2002) stages I, II, and III [9]. Interestingly, in the present study, the subgroup of surgically treated patients who had histopathological assessments including lymph node sampling, MAX_imaging,_ and lymph node metastases (histologically verified) were independent predictors of survival, whereas age, MAX_histology_, microscopic depth of invasion, vascular space invasion, and histologic grade were not. Similarly, a previous study on cervical cancer patients (*n* = 245) indicated that both histological tumor size and positive lymph node metastasis (histologically verified) independently predict poor outcome [8].

We found that in patients in whom histological tumor size measurements were available (*n* = 212), the mean MAX_histology_ was 1.5 mm larger than MAX_imaging_. Some previous studies also reported that tumor size measurements from histopathological specimens were slightly larger (~ 1–4 mm) than that based on MRI [32–34], suggesting this to be due to post-surgical change in tumor shape. Thus, tumor size measurements from MRI and histopathology may not necessarily be identical or yield the same prognostic power or optimal cutoffs for prognostication. However, in the present study, we found good overall agreement between MRI tumor size and histopathology measurements (ICC: 0.77) and similar AUCs of MAX_imaging_ (AUC = 0.83) and MAX_histology_ (AUC = 0.76) for predicting disease-specific survival, suggesting that the prognostic power of tumor size derived from histology and MRI is relatively similar.

MRI-assessed tumor volume (TVOL_imaging_) yielded an AUC of 0.84 for predicting disease-specific survival at 3 years in the present study. A previous study (*n* = 106) also reported tumor volume to yield a high AUC (AUC = 0.91 using standard ROC) for predicting disease-specific survival at 3 years [15]. Their slightly higher AUC may be partly explained by differences in ROC analyses since we used a time-dependent ROC analysis, regarded as more appropriate for survival data since the estimators in this method account for censored cases [35].

We found an excellent inter-reader agreement for the different MRI-derived tumor size measurements with ICCs of 0.83–0.85. Moreover, analyses restricted to cases with visible tumors still yield good but slightly lower ICC values (0.69–0.73). Similarly, excellent agreement with an ICC of 0.92 was reported for maximum tumor size measurements at MRI by three readers in a previous study on cervical cancer patients (*n* = 110) [17]. Interestingly, almost identical inter-reader reproducibility metrics (ICCs of 0.78–0.85) have been reported for MRI-based tumor size measurements in endometrial cancer [36], illustrating the robustness of pelvic MRI for a conspicuous depiction of tumor boundaries in all uterine cancers. Importantly, high reproducibility for MRI-based tumor measurements in cervical cancer also supports its incorporation into the 2018 FIGO staging system.

This study has some limitations. The MRI examinations for the patients in this study were performed at different hospitals, using varying scanners and imaging protocols as part of the routine primary diagnostic work-up. Although this lack of standardization of imaging protocols may have affected our results, it could be argued that the strong prognostic power of tumor size demonstrated in this study is more likely to be translatable to a standard diagnostic setting for cervical cancer, where variations in scanners and protocols do exist. Furthermore, this study has a retrospective design with MRI scans acquired during 2002–2017, implying that older scanner technologies were used in the first time period. However, since advances in MRI technologies during the last decades have improved soft tissue resolution and overall imaging quality, it is likely that the inter-reader reproducibility for tumor size measurements would have been even higher if all imaging examinations were from modern MRI scanners.

In summary, all MRI-derived tumor size measurements were strong predictors of disease-specific survival in cervical cancer, but only maximum diameter had an independent impact on survival when including all tumor size variables in the model. Maximum diameter yielded high discriminatory performance for predicting long-term disease-specific survival in cervical cancer, with maximum diameter ≥ 4.0 cm as the optimal cutoff. Furthermore, the inter-reader reproducibility for maximum diameter was excellent, supporting the incorporation of MRI-assessed maximum tumor size into the 2018 FIGO staging system in cervical cancer. Importantly, MRI-based tumor measurements should be explored in combination with other established biomarkers in cervical cancer to identify how tumor size should be used in combination with other markers to better guide risk-stratified primary treatment and follow-up algorithms in cervical cancer.

## Supplementary Information

**Additional file 1. Figure S1.** Time-dependent receiver operating characteristic curves (tdROC) at 5 years after diagnosis for predicting disease-specific survival (DSS) based on MAXimaging and MAXhistology in 212 patients. MAXimaging and MAXhistology yielded similar areas under the tdROC curves (AUC = 0.83 and 0.76, respectively) (*p* = 0.38).

**Additional file 2. Table S1: **Comparison of patients with cervical cancer from theentire patient cohort and the MRI study cohort.** Table S2: **Inter-reader reproducibility for MRI tumor size measurements by 3 readers inpatients with cervical cancer.** Table S3: **Uni- and multivariable hazard ratios for MRI-derived tumor size variables forpredicting progression- or recurrence-free survival in 416 patients with uterine cervical cancer (89patients had progression or recurrence).

## Data Availability

The data used in this study are not publicly available, as it comprises sensitive patient data. Fully anonymized data may be shared by the corresponding author upon reasonable request.

## Abbreviations

AP_imaging_: Largest anteroposterior tumor diameter in the axial oblique plane on MRI
AUC: Area under the curve
FIGO: International Federation of Gynecology and Obstetrics
IAUC: Integrated area under the curve
MAX_histology_: Maximum tumor diameter in histological samples
MAX_imaging_: Maximum tumor diameter (irrespective of plane) on MRI
SAG_imaging_: Largest tumor diameter parallel to the long axis of the cervical lumen in sagittal plane on MRI
tdROC: Time-dependent receiver operating characteristics curve
TV_imaging_: Largest transverse tumor diameter in the axial oblique plane on MRI
TVOL_imaging_: Calculated tumor volume on MRI
