# Supplementary figures and images for: What MRI-based tumor size measurement is best for predicting long-term survival in uterine cervical cancer?
Source: Insights Imaging. 2022 Jun 17;13:105. doi: 10.1186/s13244-022-01239-y (PMC9206052; doi:10.1186/s13244-022-01239-y)

Suppl. figure 1

DSS at 5 years

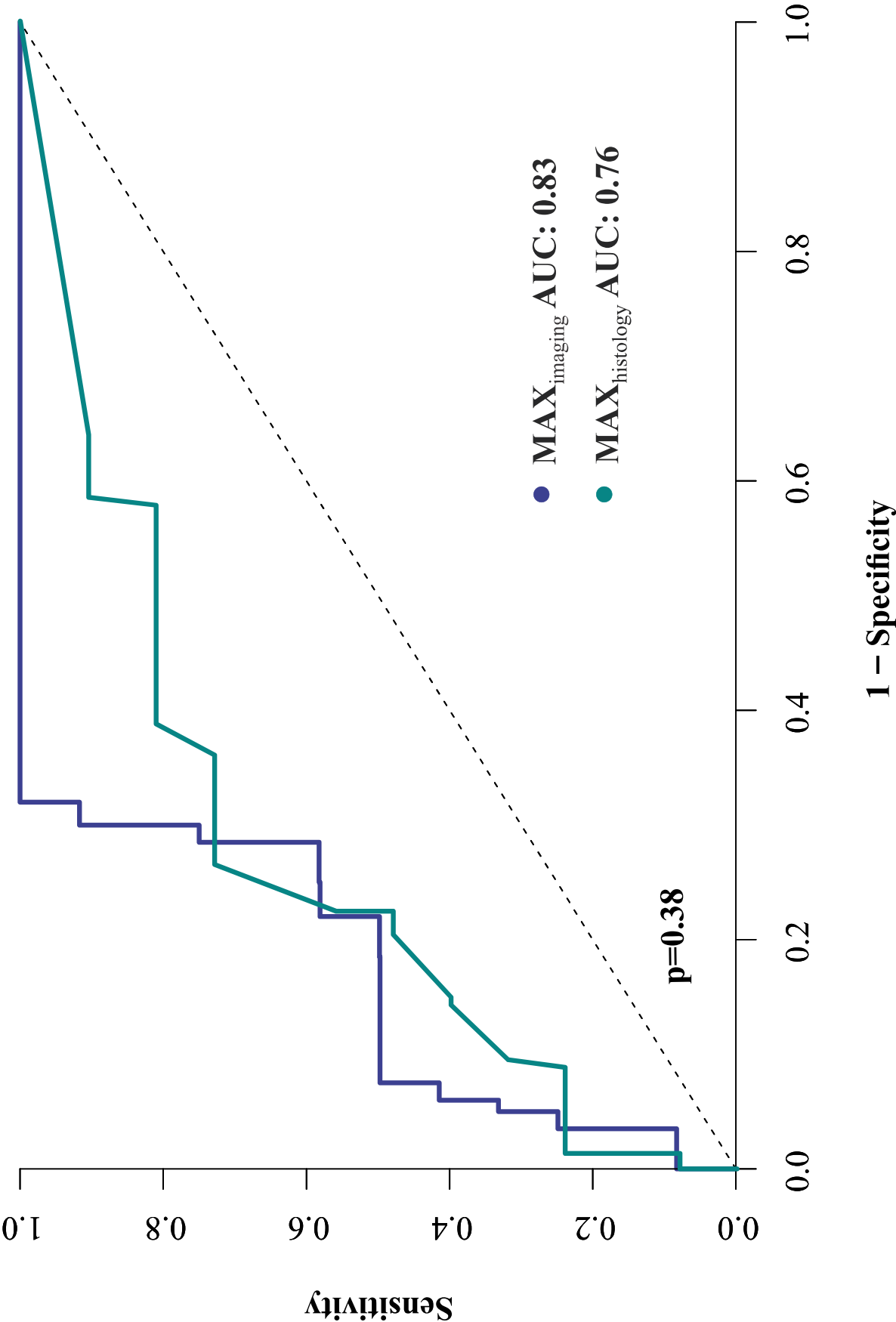

Supplement: Supplementary file 1 — Additional file 1. Figure S1. Time-dependent receiver operating characteristic curves (tdROC) at 5 years after diagnosis for predicting disease-specific survival (DSS) based on MAXimaging and MAXhistology in 212 patients. MAXimaging and MAXhistology yielded similar areas under the tdROC curves (AUC = 0.83 and 0.76, respectively) (p = 0.38). [file 13244_2022_1239_MOESM1_ESM.pdf]
