# Supplementary material for: What MRI-based tumor size measurement is best for predicting long-term survival in uterine cervical cancer?
Source: Insights Imaging. 2022 Jun 17;13:105. doi: 10.1186/s13244-022-01239-y (PMC9206052; doi:10.1186/s13244-022-01239-y)
Supplement: Supplementary file 2 — Additional file 2. Table S1: Comparison of patients with cervical cancer from theentire patient cohort and the MRI study cohort. Table S2: Inter-reader reproducibility for MRI tumor size measurements by 3 readers inpatients with cervical cancer. Table S3: Uni- and multivariable hazard ratios for MRI-derived tumor size variables forpredicting progression- or recurrence-free survival in 416 patients with uterine cervical cancer (89patients had progression or recurrence). [file 13244_2022_1239_MOESM2_ESM.pdf]

Suppl. Table 1: Comparison of patients with cervical cancer from the entire patient cohort and the MRI study cohort

Variable	Whole cohort (n_1=724)	MRI cohort (n_2=416)	p
**Age (yrs.)**			0.78*
Median (IQR)	43 (36–55)	43 (36–55)	
**Current/previous smoker (n_1=707, n_2=411)**			0.16**
No	451	275	
Yes	253	127	
**FIGO (2009) stage (n_1=722, n_2=416)**			0.30**
I	527	282	
II	118	80	
III	50	37	
IV	27	17	
**Clinical tumor size (cm) (n_1=379, n_2=230)**			0.41**
<2	93	46	
2–4	174	109	
>4	112	75	
**Primary treatment (n_1=724, n_2=416)**			0.002**
Surgery only	395	210	
Surgery and adjuvant treatment	100	51	
Primary radiotherapy with or without chemotherapy	193	147	
Other	36	8	
**Microscopic depth of invasion (mm) (n_1=344,n_2=181)**			0.71*
Median (IQR)	6 (3–9)	6 (3–9)	
**Histologic subtype (n_1=724, n_2=416)**			0.78**
Adenocarcinoma	160	92	
Squamous cell carcinoma	512	292	
Other	52	32	
**Histologic grade (n_1=582, n_2=343)**			0.67**
Low/medium	438	253	
High	144	90	

*Mann–Whitney U test **Pearson`s chi-square test
FIGO, International Federation of Gynecology and Obstetrics; IQR, interquartile range

Suppl. Table 2: Inter-reader reproducibility for MRI tumor size measurements by 3 readers in patients with cervical cancer .

	Mean (cm)			Mean difference (IQR) (cm)			ICC (95% CI)
	reader 1	reader 2	reader 3	reader 1/2	reader 1/3	reader 2/3	
All patients (n=416)							
$AP_{imaging}$	2.1	2.1	2.2	0 (0.9)	0.1 (1.3)	0 (1.2)	0.84 (0.82-0.87)
$TV_{imaging}$	2.2	2.4	2.5	0.3 (0.9)	0.3 (1.2)	0 (1.3)	0.85 (0.83-0.88)
$SAG_{imaging}$	2.0	2.3	2.5	0.3 (1.0)	0.5 (1.6)	0.2 (1.6)	0.83 (0.80-0.85)
$MAX_{imaging}$	2.9	2.9	3.0	0 (1.2)	0 (1.8)	0 (1.7)	0.85 (0.83-0.87)
Patients with visible tumor only (n=270)							
$AP_{imaging}$	3.2	3.2	3.3	0 (0.6)	0.1 (0.6)	0.1 (1.0)	0.69 (0.64–0.74)
$TV_{imaging}$	3.3	3.6	3.7	0.4 (0.7)	0.5 (0.9)	0.1 (1.0)	0.70 (0.64–0.74)
$SAG_{imaging}$	3.0	3.5	3.8	0.5 (0.8)	0.8 (1.4)	0.5 (1.2)	0.73 (0.68-0.77)
$MAX_{imaging}$	4.5	4.4	4.5	0 (0.6)	0 (1.0)	0 (1.1)	0.72 (0.67-0.76)

$AP_{imaging}$, anteroposterior diameter; ICC, intraclass correlation coefficient; IQR, interquartile range; $MAX_{imaging}$, maximum diameter; $SAG_{imaging}$, sagittal diameter; SD, standard deviation; $TV_{imaging}$, transverse diameter

Suppl. Table 3: Uni- and multivariable hazard ratios for MRI-derived tumor size variables for predicting progression- or recurrence-free survival in 416 patients with uterine cervical cancer (89 patients had progression or recurrence)

**Variables**	**Univariable HR (95% CI)**	**p***	**Multivariable HR (95% CI)†**	**p***
$AP_{imaging}$ (cm)	1.70 (1.53–1.88)	**<0.001**	1.17 (0.84–1.64)	0.71
$TV_{imaging}$ (cm)	1.61 (1.47–1.77)	**<0.001**	1.05 (0.78–1.40)	0.76
$SAG_{imaging}$ (cm)	1.39 (1.31–1.48)	**<0.001**	0.83 (0.67–1.04)	0.34
$MAX_{imaging}$ (cm)	1.41 (1.33–1.50)	**<0.001**	1.47 (1.11–1.95)	**0.03**
	**Univariable HR (95% CI)**	**p***	**Multivariable HR (95% CI)†**	**p***
$MAX_{imaging}$ (cm)	1.41 (1.33–1.50)	**<0.001**	1.26 (1.17–1.36)	**<0.001**
Age (decade)	1.53 (1.36–1.73)	**<0.001**	1.37 (1.17–1.58)	**<0.001**
FIGO (2018) stage (III/IV vs. I/II)	7.24 (4.69–11.19)	**<0.001**	2.85 (1.72–4.73)	**<0.001**
**$MAX_{imaging}$ (cm) in FIGO (2018) subgroups**	**Univariable HR (95% CI)**	**p***	-	-
FIGO I (n=260)	1.66 (1.28–2.16)	**<0.001**	-	-
FIGO II (n=50)	1.18 (0.85–1.63)	0.56	-	-
FIGO III (n=81)	1.29 (1.14–1.47)	**<0.001**	-	-
FIGO IV (n=25)	1.09 (0.93–1.27)	0.56	-	-
**Subgroup analysis of surgically treated patients who had histopathological assessments of primary tumor (n=261; 26 patients had progression or recurrence) with pelvic lymph node sampling (n= 200 patients; 23 patients had progression or recurrence)‡**				
	**Univariable HR (95% CI)**	**p***	**Multivariable HR (95% CI)♀**	**p***
$MAX_{imaging}$ (cm)	1.46 (1.27–1.67)	**<0.001**	1.41 (1.25–1.59)	**<0.001**
Age (decade)	1.42 (1.07–1.88)	**0.03**	-	-
$MAX_{histology}$ (cm)	1.34 (1.20–1.50)	**<0.001**	-	-
Inflammatory reaction (yes vs. no)	0.75 (0.25–2.22)	0.60	-	-
Microscopic depth of invasion (cm)	3.78 (1.78–8.04)	**0.007**	-	-
Vascular space invasion (yes vs. no)	4.40 (2.04–9.52)	**0.005**	-	-
Histologic grade (high vs. low/medium)	4.73 (2.14–10.43)	**<0.001**	-	-
Lymph node metastasis (yes vs. no)	7.15 (3.23–15.79)	**<0.001**	5.70 (2.58–12.60)	**<0.001**

$AP_{imaging}$, anteroposterior tumor diameter at MRI; CI, confidence interval; FIGO, International Federation of Gynecology and Obstetrics; HR, hazard ratio; $MAX_{imaging}$, maximum tumor diameter at MRI; $MAX_{histology}$, maximum tumor diameter in histological samples; MRI, magnetic resonance imaging; $SAG_{imaging}$, sagittal tumor diameter at MRI; $TV_{imaging}$, transverse tumor diameter at MRI

*Cox proportional-hazards model; all p values corrected for multiple testing with Holm−Bonferroni method. Significant p-values are given in boldface.

†Includes all variables listed

‡Missing data were handled by multiple imputation in order to perform multivariable analysis on all patients treated with surgery

♀Variables in model were selected by using the "fastbw"-function in the "rms" r-package (1)
